# Cell Signaling with Extracellular Thioredoxin and Thioredoxin-Like Proteins: Insight into Their Mechanisms of Action

**DOI:** 10.1155/2017/8475125

**Published:** 2017-09-12

**Authors:** Thierry Léveillard, Najate Aït-Ali

**Affiliations:** ^1^INSERM, U968, Sorbonne Universités, 75012 Paris, France; ^2^UPMC Univ Paris 06, UMR_S 968, Institut de la Vision, 75012 Paris, France; ^3^CNRS, UMR_7210, 75012 Paris, France

## Abstract

Thioredoxins are small thiol-oxidoreductase enzymes that control cellular redox homeostasis. Paradoxically, human thioredoxin (TXN1) was first identified as the adult T cell leukemia-derived factor (ADF), a secreted protein. ADF has been implicated in a wide variety of cell-to-cell communication systems acting as a cytokine or a chemokine. TRX80 is a truncated TXN1 protein with cytokine activity. The unconventional secretion mechanism of these extracellular thioredoxins is unknown. The thioredoxin system is relying on glucose metabolism through the pentose phosphate pathway that provides reducing power in the form of NADPH, the cofactor of thioredoxin reductase (TXNRD). While a complete extracellular TXN system is present in the blood in the form of circulating TXN1 and TXNDR1, the source of extracellular NADPH remains a mystery. In the absence of redox regenerating capacity, extracellular thioredoxins may rather be prooxidant agents. Rod-derived cone viability factor (RdCVF) is the product of intron retention of the nucleoredoxin-like 1 (*NXNL1*) gene, a secreted truncated thioredoxin-like protein. The other product encoded by the gene, RdCVFL, is an enzymatically active thioredoxin. This is a very singular example of positive feedback of a superthioredoxin system encoded by a single gene likely emerging during evolution from metabolic constraints on redox signaling.

## 1. Introduction

The inverse correlation between the life-span and the rate of oxygen consumption of mammals has directed research on aging into the field of oxygen metabolism. Reactive oxygen species (ROS), which are too reactive to exist in biological systems, are formed *in situ* by leakage from the mitochondrial respiratory chain generating cumulative cellular dysfunctions. ROS are continuously produced in the cell as a product of aerobic life. In order to avoid or to reverse the damage to macromolecules by ROS, proper redox conditions must be maintained within the intracellular environment. Therefore, aerobic organisms have several antioxidant systems including superoxide dismutase, catalase, and thioredoxin (TXN) systems to compensate for this inherent fragility. The prototype of the thioredoxin proteins, TXN1, is a 12 kDa protein with the redox-active disulfide/dithiol group within the conserved active-site sequence CGPC. Reduced TXN1 catalyses the reduction of disulfide bonds in many cytoplasmic and nuclear proteins, and oxidized TXN1 is reversibly reduced by the action of the thioredoxin reductase using electron transfer from nicotinamide adenine dinucleotide phosphate (NADPH) [1]. The TXN system is compartmentalized into distinct steady-state redox potentials within each cellular organelle.

Cysteine is a rarely used amino acid that accounts for about 2% of the amino acids in eukaryotic proteins. ROS, as well as reactive nitrogen species (RNS), can induce redox signals by means of oxidative modifications of cysteine residues in targeted proteins. ROS and RNS, including hydrogen peroxide (H_2_O_2_), superoxide ion (O_2_^•−^), nitric oxide (NO^•^), and hydroxyl radical (OH^•^), can be produced *in vivo* from a wide range of cellular processes. The large, polarizable sulfur atom in a thiol group is electron rich and highly nucleophilic; hence, cysteines can undergo a broad range of chemical reactions [2]. C-SH is in equilibrium with C-S^−^ and to the disulfide S-S, which can be oxidized by ROS to C-SOH, -SO_2_H, and SO_3_H, or S-nitrosylated by RNS to C-SNO and finally, in the presence of glutathione (GSH), S-thiolated to -S-SG. Cysteines differ in their reactivity properties depending of the protein microenvironment [3]. As many of these modifications are reversible through reduction catalyzed by oxidoreductases, such as thioredoxins (TXNs), glutaredoxins (GRXs), sulfiredoxin (SRX), and sestrins (SESs), protein thiol redox state can respond to the redox environment.

Originally, human TXN1 was identified as an extracellular protein which is extending the field of redox biology to the functions of thioredoxins in a compartment that may not be under the control of the cellular TXN reducing system [4]. In order to develop this question in a comprehensive manner, we have chronologically reviewed key findings on extracellular thioredoxins.

## 2. Adult T Cell Leukemia-Derived Factor at the Origin of Extracellular Thioredoxins

**Figure 1:**
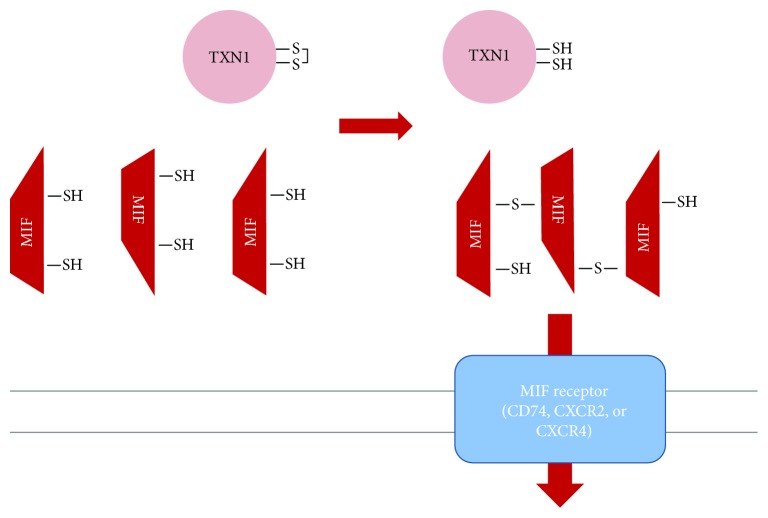
Hypothetical model for ADF/TXN1 extracellular signaling based on MIF trimerisation. The cytokine effect of ADF/TXN1 mediated through MIF cell surface receptors.

Adult T cell leukemia (ATL) is the first human cancer found to be caused by a retrovirus [5]. ATL arises in ~1% of human T cell lymphotropic virus type 1- (HTLV1-) infected individuals after a latent period of 10 to 20 years [6]. ATL cell lines produce a soluble factor that stimulates the expression of interleukin-2 receptor alpha chain (IL2RA) by HTLV1-positive/ATL-negative cells, the ATL-derived factor (ADF) [7]. The activity was purified to homogeneity by four successive chromatographic steps from the conditioned medium of an ATL cell line (ATL-2). The N-terminal sequence obtained by Edman degradation was used to isolate a cDNA encoding a 105 amino acid protein by screening a cDNA library constructed with ATL-2 cells [4]. ADF is homologous to the bacterial thioredoxin TXN1 and identical to human TXN1. The same extracellular thioredoxin was identified in the conditioned medium of 3B6 cells from an Epstein-Barr virus- (EBV-) infected B-lymphoblastoid cell line [8]. Recombinant ADF/TXN1 enhances the cytokine effect of interleukin (IL) 1 and IL2, suggesting that ADF might sensitize these cells to a specific subset of interleukins. The potentiation of ADF/TXN1 biological activity by 2-mercaptoethanol, a reducing agent, suggests that cell signaling involves the reduction of an unknown ADF/TXN1 cell surface protein. The authors proposed at that time (1989) that using radiolabeled recombinant ADF/TXN1 could clarify the nature of the cell surface-binding molecules. Twenty-eight years later, the result of such experiment is still awaited [9]. Protein purification, while conducted to homogeneity, is not a guarantee that the identified peptide is necessarily carrying the biological activity. Current proteomic methods might have revealed the existence of many other proteins in the most purified ADF/TXN1 fraction. This remark emphasizes that even experts in protein purification are not immune to overinterpretations. The conditioned medium of COS cells transfected with ADF/TXN1 cDNA replicates the growth-promoting activity of that of ATL-2 cells, but COS cells might also secrete IL1, IL2, or other cytokines whose action may be enhanced by oxidoreduction of their cysteines. In fact, following the purification of ADF/TXN1 by four chromatographic steps, two peptides A and B were identified. Peptide A was used to identify ADF/TXN1, and later on, peptide B led to the identification of macrophage migration inhibitory factor (MIF) [10]. MIF binds to ADF/TXN1, and both form a complex in the conditioned medium of ATL-2 cells [11]. Internalization of extracellular MIF in ATL-2 cells is facilitated by ADF/TXN1. The activity of ADF/TXN1 could theoretically be relayed by CD74, C-X-C chemokine receptor type 2 (CXCR2), or CXCR4, one of the cell surface receptors of MIF [12, 13]. MIF is a cytokine with two distinct catalytic activities, tautomerase/isomerase and thiol-oxidoreductase activities [14]. The cytokine activity of MIF requires the formation of a homotrimeric complex through interdisulfide bond formation [15]. Ebselen, a MIF inhibitor, forms a covalent bond with MIF cysteine 80, which led to dissociation of trimers to monomers and the loss of cytokine activity [16]. Overall, these results suggest an alternative mechanism for which, besides the existence of a TXN1 cell surface receptor, TXN1 might regulate enzymatically the trimerisation of MIF and thus its biological activity through one of the MIF cell surface receptors (Figure 1).

Notwithstanding, *a bona fide* TXN1 cell surface receptor, the tumor necrosis factor receptor superfamily member 8 (TNFRSF8/CD30), was identified [17]. A mutant TXN1 protein lacking the resolving cysteine of the catalytic site (CXXC > CXXS), resulting in the formation of a stable intermediate between mutant TXN1 and its substrate, was used as a probe. This mutant trapped cell surface proteins from the EBV-transformed lymphoblastoid B-cell line, a previously identified target of ADF/TXN1 [8]. Competition analysis with TNFRSF8 antibodies suggested that ADF/TXN1 induces a redox-dependent conformational change within the extracellular domain of TNFRSF8. Only a partial conformation rearrangement was observed when reduced TXN1/ADF was applied in the absence of thioredoxin reductase and NADPH confirming that the enzymatic reaction requires the regeneration of reduced ADF/TXN1, oxidized after TNFRSF8 thiol-oxidoreductase reaction [18]. Surprisingly, proliferation assays mediated by TNFRSF8 of the EBV-transformed lymphoblastoid B-cell line have not been reported since 2007 [8]. Recombinant ADF/TXN1 also activates the short transient receptor potential channel 5 (TRPC5) by means of a similar thiol-oxidoreductase mechanism [19].

## 3. The Paradox of the Redox Power of Extracellular Thioredoxins

**Figure 2:**
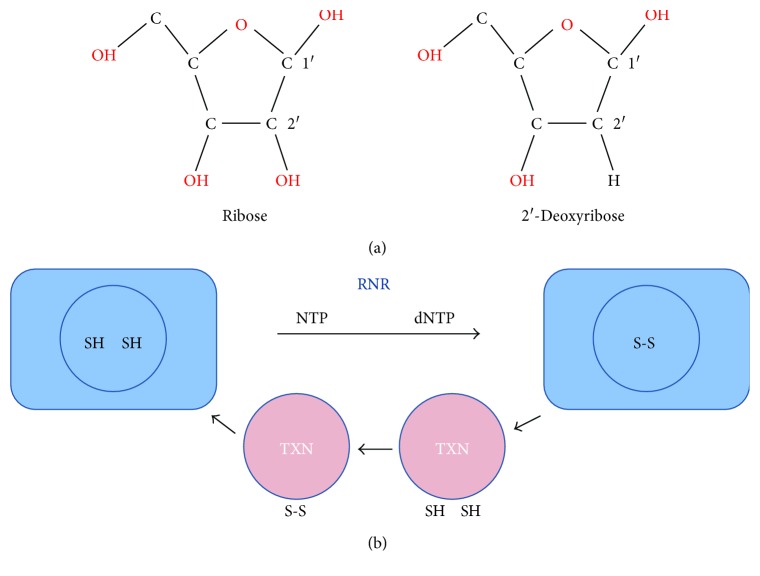
Deoxyribonucleotide by ribonucleotide reductase. (a) Chemical structure of ribose and 2′deoxyribose. (b) Ribonucleotide reductase catalysis and recycling. RNR: ribonucleotide reductase, NTP: ribonucleotide triphosphate, dNTP: deoxyribonucleotide triphosphate, TXN: thioredoxin.

Deoxyribonucleotides are all made from a ribonucleotide precursor by the action of ribonucleotide reductase (RNR), a tetramer of two subunits, R1 and R2 (Figure 2(a)). During ribonucleotide reductase catalysis, a cysteinyl radical is formed. After the completion of one turnover cycle, a disulfide bond is formed between the conserved cysteine pairs at the R1 active site which is transferred at a C-terminal dithiol through disulfide exchange. Then, the resulting disulfide bond at the C-terminal tail is reduced by the thioredoxin (TXN) system reactivating R1 for the next cycle of RNR catalysis [20] (Figure 2(b)). So the reduction of ribonucleotides, the rate-limiting step of the DNA synthesis, depends on the reducing power provided by NADPH through thioredoxin enzymes. TXN is ubiquitously distributed from archaea, bacteria, plants, and animals [21]. *E. coli* thioredoxin 1 (TRXA) was originally identified in 1964 by the group of Laurent et al. as a protein required for ribonucleotide reduction by ribonucleotide reductase [22]. The term thioredoxin refers to the biological function of a protein that depends on the cyclic reduction-oxidation of a single S-S group of the compound. Holmgren then disclosed the 109-amino acid sequence of TRXA and characterized the two cysteine residues (C32 and C35) of the catalytic site [23]. Years of intense research in this field revealed the dependence of the thioredoxin system on glucose metabolism through the pentose phosphate pathway that provides reducing power in the form of NADPH, the cofactor of thioredoxin reductase (TXNRD) [24]. How are oxidized extracellular thioredoxins regenerated in the rather oxidized extracellular compartment? [25]. A complete extracellular TXN system is present in the blood in the form of circulating TXN1 and TXNDR1. The procoagulant activity of cell surface tissue factor (F3), a member of the cytokine receptor family involved in the onset of coagulation, is negatively regulated by the TXN1 system that reduces F3 cysteines 186 and 209 [26]. Interestingly, the process is sensitive to change in the ratio NADPH/NADP^+^ which dictates the redox states and activity of TXN1/TXNRD1. The origin of extracellular NADPH was not revealed by that study and remains mysterious since NADPH is not transported across intracellular membranes [27]. In the absence of NADPH and TXNRD, extracellular thioredoxins will not act as enzymes [28]. The catalytic site of reduced thioredoxins could become oxidized (SH, SH > S-S) while oxidized thioredoxins could become reduced (S-S > SH, SH), but those reactions are irreversible. The exchanged electrons would play here the role of a one-shot biological signal.

## 4. TRX80, a Proteolytic Product of Thioredoxin 1 Involved in Cell-to-Cell Communication

**Figure 3:**
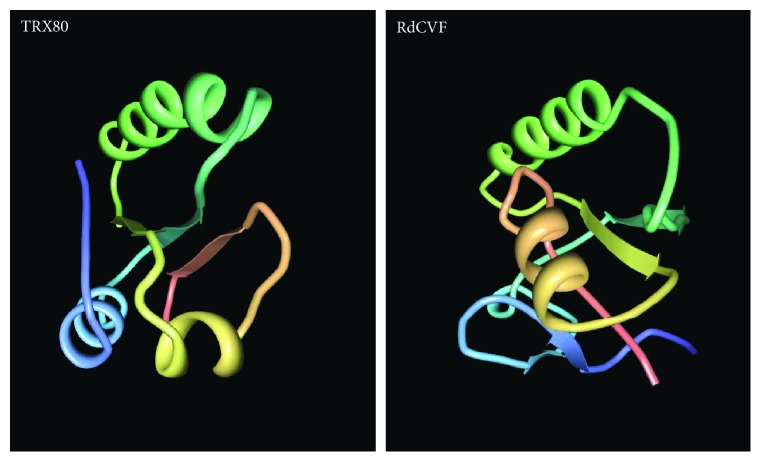
Structure models of TRX80 and RdCVF based on the X-ray structure of human TXN1 for TRX80 and on that of the tryparedoxin TRYX of *Crithidia fasciculata*.

In addition to its cytokine activity, TXN1 is a chemoattractant for granulocytes, monocytes, and T-lymphocytes with a range of activities similar to other classical chemokines [29]. TXN1/ADF released from HTLV-1-infected cells carries this chemotactic activity. The mutant TXN1/ADF protein lacking the resolving cysteine of the catalytic site (CXXC > CXXS) is not active as a chemokine, showing that the redox function of TXN1/ADF is involved. It is presently not yet known if the chemokine and the cytokine activities of TXN1/ADF rely broadly or partly on the same downstream signaling molecules [9]. Through an independent research direction, a heterogeneous cytotoxic activity for the subclass of eosinophilic granulocytes was isolated from the conditioned medium of monocytes. It comprises two distinct forms of TXN1, a 14 and a 10 kDa peptide [30]. TXN1 (10 kDa) is 20-fold more active than TXN1 (14 kDa) in biological assays. The 10 kDa protein corresponds to the 80–84 N-terminal amino acids of TXN1 and was then named TRX80 accordingly [31]. TRX80 cannot be reduced by TXNRD and NADPH most likely because the missing C-terminal sequence is required for the interaction with TXNRD1 or alternatively because the truncation within the thioredoxin fold in TRX80, removing one strand (84–92) and one alpha helix (93–105), impairs with the overall 3D structure involved in the interaction [32]. Without the regenerative action of the TXNRD and NADPH, TRX80 cannot sustain thiol-oxidoreductase enzymatic activity. Interestingly, TRX80 catalytic cysteines can be reduced by TXN1 in a TXNRD1/NADPH-dependent manner. In that configuration, TRX80 is a target of TXN1. TRX80 is a truncated thioredoxin that shares similarities with another truncated thioredoxin, the rod-derived cone viability factor (RdCVF) [33] (Figure 3). The correlation between the induction of expression of TRX80 and that of metalloproteinases ADAM10 and ADAM17 by phorbol 12-myristate 13-acetate (PMA) [30, 34] led to the discovery that TRX80 is produced from TXN1 by cleavage in monocytes by the alpha-secretases ADAM10 and ADAM17 [35]. The biological functions of extracellular TRX80 is quite diverse; originally cytotoxic, it was shown to act as a cytokine for monocytes, to promote proinflammatory macrophage phenotype, to inhibit beta-amyloid peptide aggregation, and to activate the classical and alternative pathways of complement activation [35–38]. Importantly, here, the mitogenic cytokine effect on peripheral blood mononuclear cells does not involve any thiol-oxidoreductase activity provided by TRX80 [39]. It seems that the TXN1 sequence corresponding to TRX80 was recruited by the immune system to exert cell communication functions that lost their relation to redox biology.

## 5. Thioredoxin Secretion, a Long Lasting Mystery

Thioredoxins are secreted but have no N-terminal signal sequence and consequently are not secreted through the endoplasmic reticulum and Golgi pathway but by mechanisms that are referred to as unconventional protein secretion pathways [40]. The mechanism of secretion of thioredoxins has remained a mystery for more than 25 years after its first observation and is even currently ignored [41]. The four principal types of unconventional protein secretion can be divided into nonvesicular and vesicular pathways. The nonvesicular pathways encompass self-sustained protein translocation across plasma membranes (type I, FGF2) and ABC-transporter-based secretion (type II, yeast pheromone a-factor). Vesicular pathways are characterized by autophagy-based secretion and secretory lysosomes (type III, IL1*β* or IL1B) and proteins that bypass the Golgi complex for trafficking to the plasma membrane (type IV, CFTR). The type of secretory mechanism of thioredoxins remains unknown [4, 40]. TXN1 secretion *in vitro* is temperature sensitive and inhibited by unknown factors present in serum [42]. Its secretion appears to be mediated by a pathway distinct from IL1B as TXN1 could be detected neither in intracellular vesicles nor in its secretion blocked by ABC transporter inhibitors. However, even if controversial, secretion of thioredoxin is inhibited by methylamine, a lysosome inhibitor, as for that of IL1B [42]. Surprisingly, the redox state of TXN1 does not influence its unconventional export [42].

## 6. The Truncated Thioredoxin Rod-Derived Cone Viability Factor Links Redox Homeostasis to Glucose Metabolism

The retina of vertebrates is dual with rod photoreceptors for dim-light vision and cone photoreceptor for color and daylight vision [43]. They differentiate by the morphology of the outer segment made of stacks of membranal disks or invaginations containing the light-sensitive molecule as originally observed by Schultze in 1866 [44]. The truncated thioredoxin rod-derived cone viability factor (RdCVF) was identified by high content screening. A retinal cDNA library from a wild-type mouse was used as pools of 100 plasmids to transfect COS-1 cells. Conditioned media harvested from these cells were incubating with a cone-enriched culture prepared from the retina of chicken embryos in the absence of serum [45]. In this culture, progenitor cells isolated from chicken embryos at embryonic day 6 [stage 29] were platted at low density and differentiated into cones. The viability of cells after 7 days *in vitro* was used as the readout of the assay. The set-up of the screening implies that the active polypeptide would be secreted by COS-1 cells. One pool out of the 2100 screened was diluted and led to a unique clone encoding for an open reading frame of a putative polypeptide of 109 amino acids, the RdCVF protein [46]. RdCVF was further shown to protect cones from the mouse. Retrospectively, the thought that the RdCVF factor could not have been identified if we had realized the cone-enriched cultures in the presence of serum gives vertigo. RdCVF, an alternative splicing product of nucleoredoxin-like 1 (*NXNL1)* gene, is secreted by rods that also encodes for an active thioredoxin enzyme RdCVFL with an entire thioredoxin fold and a CXXC catalytic domain [33]. RdCVFL protects photoreceptors, rods, and cones, against oxidative damage [47–49]. Since RdCVF is truncated within the thioredoxin fold, like TRX80, it does not have enzymatic activity and consequently must signal through a cell surface receptor on cones. A far-western blotting approach using the cone-dominated retina of chicken embryos was used to identify cell surface proteins interacting with GST-RdCVF after the transfer of the proteins resolved by electrophoresis on a nitrocellulose membrane. Proteins migrating along with the specific signal that was detected were identified by mass spectrometry [50]. Among the identified proteins, basigin 1 (BSG1) is a transmembrane protein representing a splice variant with an additional third extracellular immunoglobulin domain (Ig0) of the basigin gene (*Bsg*) expressed specifically by photoreceptors. This interaction was validated in a cellular context using transiently transfected BSG1 cDNA with RdCVF-alkaline phosphatase fusion protein and a colorimetric assay. Silencing *Bsg* in chicken cone-enriched cultures reduced cell survival mediated by RdCVF indicating that the BSG1 is the RdCVF transducing cell surface receptor. Unfortunately, BSG1 was not previously known as a cell surface receptor but rather as a protein involved in cell adhesion [51]. Its intracellular domain is short and does not carry any informative motifs such as a tyrosine phosphorylation site. Therefore, we used a BSG1 antibody to coimmunoprecipitate BSG1-interacting proteins from membrane fraction of the retina of chicken embryos and identified these proteins by mass spectrometry [50]. BSG1 interacts with the glucose transporter GLUT1/SLC2A1. The formation of the complex RdCVF/BSG1/GLUT1 on cone surface stimulates glucose entry into the cones. The mechanism that leads to an accelerated glucose entry into cones via GLUT1/SLC2A1 after RdCVF binding to BSG1 is presently unknown. Considering that the catalytic site of extracellular thioredoxins could become oxidized (SH, SH > S-S), RdCVF could act as a prooxidant that triggers the conversion of homodimeric and reduced form of GLUT1/SLC2A1 to its homotetrameric and oxidized form that is known to transport glucose more efficiently [52]. RdCVF would be a positive allosteric heterotrophic effector of the glucose transporter GLUT1/SLC2A1 [53]. Notice that TXN1/ADF is also acting as a prooxidant for MIF in the hypothetical model proposed above (Figure 1). Glucose is used by cones to produce lactate by aerobic glycolysis. The idea that cones use this particular metabolism of glucose where the pyruvate produced by glycolysis is not transported to the mitochondria, but rather metabolized to lactate by lactate dehydrogenase, is usually restricted to cancer cells and named the Warburg effect [54, 55]. This peculiar use of glucose by cones was demonstrated by measuring the oxygen consumption and extracellular acidification resulting from lactate secretion. Inhibition of pyruvate transport to the mitochondria or pentose phosphate pathway does not affect the activity of RdCVF, while, oxamate, a lactate dehydrogenase inhibitor, abolishes RdCVF effects on cones. Quite interestingly, in 1924, Otto Warburg established that aerobic glycolysis is specific of cancer cells, he also reported that avian retina metabolizes glucose by aerobic glycolysis, but he suspected at this time that this was an artifact. So 91 years have been necessary to resolve the enigma of aerobic glycolysis in the retina of cone-dominated species such as the chicken and pigeon [56]. Glucose is partially metabolized to glycerol-3-phosphate, a molecule entering the composition of phospholipids forming the membranes of cone outer segments. Phospholipids are composed of two fatty acids, a glycerol unit and a phosphate group. Fatty acids derived from food are activated in the form of acyl-CoA intermediates and esterified with glycerol-3-phosphate into phospholipids through the Kennedy pathway [57]. Glycerol-3-phosphate is made from a glycolytic metabolite, dihydroxyacetone phosphate (DHAP), the breakdown of one molecule of fructose-1,6-bisphosphate (C6) by aldolase A. Aldolase produces one molecule of glyceraldehyde-3-phosphate (C3) and one molecule of DHAP (C3). Phosphatidic acid is synthesized through multiple enzymatic steps from glycerol-3-phosphate and fatty acyl-CoA. Phospholipid synthesis is taking place on the cytoplasmic face of the endoplasmic reticulum where the membrane-bound choline/ethanolamine phosphotransferase enzyme (CEPT1) is located. This reaction produces the phospholipids, phosphatidylcholine, or phosphatidylethanolamine.

## 7. The Nucleoredoxin-Like 1 Gene Encodes for a Superthioredoxin System

The retina of the mouse with a disruption of the *Nxnl1* gene shows signs of oxidative and photooxidative damages [47]. Using adeno-associated viral vector delivery through a ubiquitous promoter, we found that RdCVF (109 residues) protects the cones of the mouse retina but not RdCVFL (217 residues) [58]. The 109 residues of RdCVF are identical to the N-terminal region of the 217 amino acid-long RdCVFL protein since RdCVF is truncated within the thioredoxin fold. The fact that the full-length RdCVF sequence does not protect cones when delivered through a ubiquitous promoter suggests that the structure of the thioredoxin fold of RdCVF is altered by the truncation. The bifunctional gene *NXNL1*, whose expression is restricted to the retina, is composed by two coding exons framing one intron. RdCVF is produced by alternative splicing. When the intron is spliced, the gene encodes for a long product coding for the thioredoxin enzyme RdCVFL, whereas when the intron is retained and due to the presence of a conserved stop codon in the reading frame, the gene encodes for a short product coding for the truncated thioredoxin RdCVF [33]. Alternative splicing is cell specific as it occurs only in rods but not in cones of the mouse retina that express only RdCVFL [49]. When using adeno-associated viral vector delivery through a cone-specific promoter, RdCVFL (217 residues) protects the cones of the mouse retina against oxidative damage. RdCVF expressed by rods stimulates cone outer segment growth. Downstream of that metabolic signaling, RdCVFL is involved in redox homeostasis. Hence, in that sense, both products of the *NXNL1* are acting toward the same goal and have a complementary activity: protecting cones [59].

**Figure 4:**
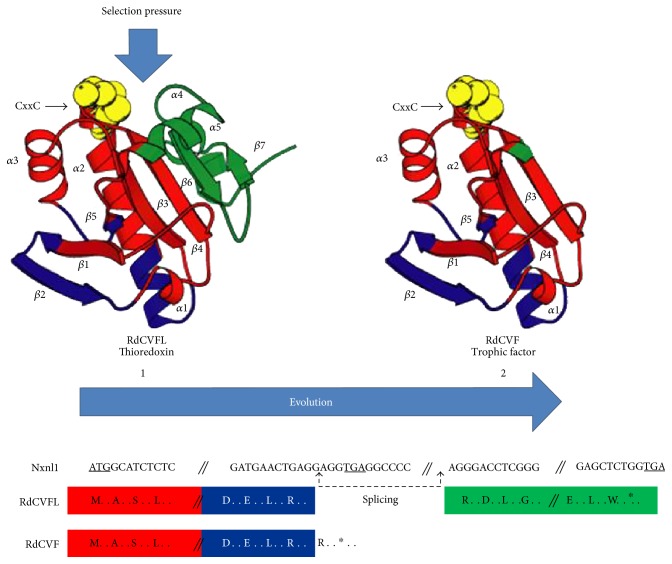
Theoretical consideration on the evolutionary history of the nucleoredoxin-like 1 gene.

**Figure 5:**
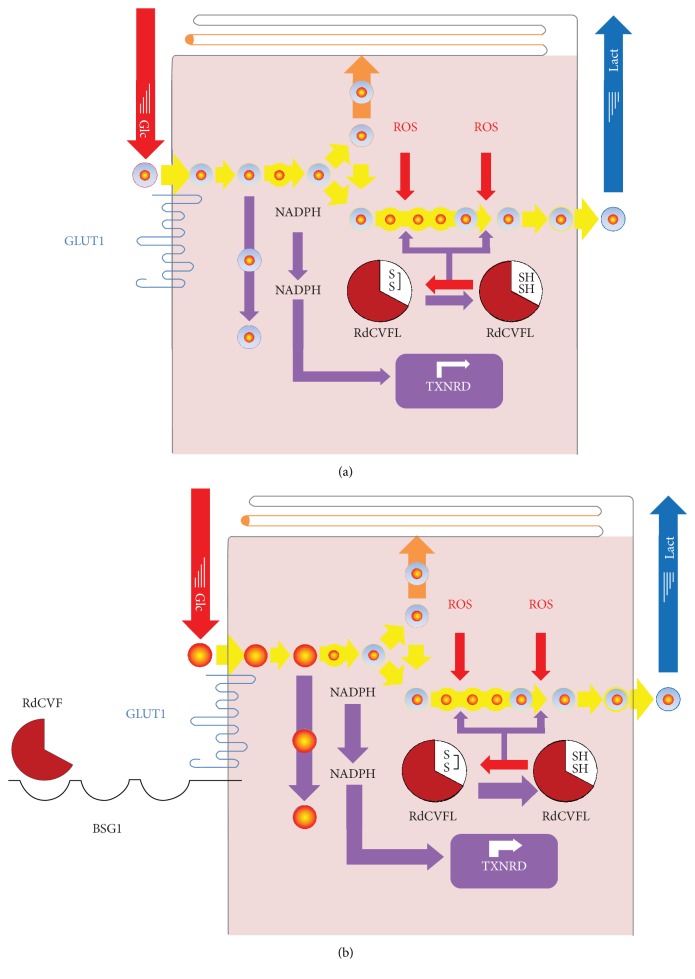
The nucleoredoxin-like 1 gene encodes for a superthioredoxin system. (a) In the absence of RdCVF signaling mediated by the complex BSG1/GLUT1, the reduction of oxidized RdCVFL in cones by thioredoxin reductase (TXNRD) and NADPH is limited by glucose uptake rate. (b) The extracellular truncated thioredoxin RdCVF accelerates glucose uptake by its interaction with the BSG1/GLUT1 complex at the surface of cones. The concentration of NADPH produced by the pentose phosphate pathway is increased. Consequently, the reduction of RdCVFL is ameliorated so that the combination of extracellular RdCVF and intracellular RdCVFL constitutes a superthioredoxin system. Glc: glucose, lact: lactate, and ROS: reactive oxygen species. Circles represent metabolites and their diameters and their concentrations. Yellow: glycolysis, Orange: triglyceride synthesis, and purple: pentose phosphate pathway.

In an evolutionary perspective, the selection pressure acts first on the thioredoxin fold of RdCVFL before acting on the truncated thioredoxin RdCVF (Figure 4). This is correlated to the fact that cones precede rods [60]. What could have been the driving force leading to the emergence of the trophic factor RdCVF? An accidental splicing leading to an extracellular truncated thioredoxin as RdCVF would not work without the expression of BSG in the form of the splicing variant having a third extracellular domain Ig0, produced by photoreceptors by alternative splicing. Are there temporal links between the appearance of the inhibition of the *NXNL1* gene's splicing leading to its intron retention and the rod appearance during evolution? Regardless of the evolution history of RdCVF signaling, it is quite reasonable to propose that the thioredoxin activity of RdCVFL precedes that of RdCVF but that conjunction of both proteins is acting as a positive feedback loop for redox homeostasis in the cones and defines a superthioredoxin system (Figure 5). Meaning that the ancestral *NXNL1* gene originally encodes for a thioredoxin enzyme and then after for another protein RdCVF boosting the activity of the first one. Whatever the details, the identification of the molecular mechanisms leading to intron retention will certainly be very informative.

## 8. A Truncated Extracellular Thioredoxin as a Therapeutic Agent for Blinding Diseases

Opsin activation by photon capture requires the sensing molecule to be embedded in a lipid bilayer of optimal fluidity made of phospholipids rich in polyunsaturated fatty acids **(**PUFA) [61]. PUFA have the tendency to oxidize and must be removed daily for maintenance of the visual system [62]. Consequently, since cones are postmitotic neurons, 10% of their outer segment is removed daily by phagocytosis of the retinal pigment epithelium and renewed from the inner segment where glucose metabolism is very active. With a prevalence of 1/5000, retinitis pigmentosa is the most common form of inherited retinal degeneration. In patients suffering from retinitis pigmentosa, the vision loss develops in two successive steps. The first clinical sign of this disease is night blindness, which is the consequence of rod degeneration due to the direct action of mutations in any of the 60 distinct genes presently known to cause the disease. This is felt as a minor handicap, and these people retain an almost normal way of life [63]. Rod degeneration leads to the loss of expression of RdCVF [64]. The subsequent loss of function of cones at the centre of the retina results in reduced central vision leading ultimately to untreatable blindness. As patients usually visit ophthalmologists when their daylight vision decreases which corresponds to the moment when cones start to be affected, preventing the death of rods will not be medically effective [65]. Treating patients by restoring the expression of the superthioredoxin system made of RdCVF and RdCVFL, while not correcting the causative gene defect, should maintain cone-mediated central vision, potentially benefiting an estimated 1.5 million people worldwide [66, 67].

## 9. Conclusion

Extracellular thioredoxins have been implicated in intercellular communication early on from the discovery of TXN1/ADF. Meanwhile, in a detailed description of its mode of action and that of the truncated form of TXN1, TRX80 remains incomplete. The extracellular truncated thioredoxin RdCVF encoded by the *NXNL1* gene is to our knowledge a unique example of a complete extracellular thioredoxin signaling system.
